# Metagenome-based characterization of the gut bacteriome, mycobiome, and virome in patients with chronic hepatitis B-related liver fibrosis

**DOI:** 10.3389/fmicb.2024.1449090

**Published:** 2024-10-25

**Authors:** Wenlin Chen, Fang Liang, Yue Zhang, Yuncheng Zhang, Jinzhen Lv, Xiande Jin, Yun Ran, Shenghui Li, Wen Sun

**Affiliations:** ^1^Department of Liver Diseases, Beijing University of Chinese Medicine Shenzhen Hospital (Longgang), Shenzhen, China; ^2^Puensum Genetech Institute, Wuhan, China; ^3^Centre for Translational Medicine, Shenzhen Bao’an Chinese Medicine Hospital, Guangzhou University of Chinese Medicine, Shenzhen, China; ^4^Key Laboratory of Health Cultivation of the Ministry of Education, Beijing Key Laboratory of Health Cultivation, School of Traditional Chinese Medicine, Beijing University of Chinese Medicine, Beijing, China

**Keywords:** chronic hepatitis B-related liver fibrosis, gut microbiome, microbial dysbiosis, gut mycobiome, gut virome, whole-metagenome sequencing

## Abstract

**Introduction:**

The gut microbiota is believed to be directly involved in the etiology and development of chronic liver diseases. However, the holistic characterization of the gut bacteriome, mycobiome, and virome in patients with chronic hepatitis B-related liver fibrosis (CHB-LF) remains unclear.

**Methods:**

In this study, we analyzed the multi-kingdom gut microbiome (i.e., bacteriome, mycobiome, and virome) of 25 CHB-LF patients and 28 healthy individuals through whole-metagenome shotgun sequencing of their stool samples.

**Results:**

We found that the gut bacteriome, mycobiome, and virome of CHB-LF patients were fundamentally altered, characterized by a panel of 110 differentially abundant bacterial species, 16 differential fungal species, and 90 differential viruses. The representative CHB-LF-enriched bacteria included members of *Blautia_A* (e.g., *B. wexlerae*, *B. massiliensis*, and *B. obeum*), *Dorea* (e.g., *D. longicatena* and *D. formicigenerans*), *Streptococcus*, *Erysipelatoclostridium*, while some species of *Bacteroides* (e.g., *B. finegoldii* and *B. thetaiotaomicron*), *Faecalibacterium* (mainly *F. prausnitzii*), and *Bacteroides_A* (e.g., *B. plebeius_A* and *B. coprophilus*) were depleted in patients. Fungi such as *Malassezia* spp. (e.g., *M. japonica* and *M. sympodialis*), Candida spp. (e.g., C. parapsilosis), and *Mucor circinelloides* were more abundant in CHB-LF patients, while *Mucor irregularis*, *Phialophoraverrucosa*, Hortaea werneckii, and *Aspergillus fumigatus* were decreases. The CHB-LF-enriched viruses contained 18 *Siphoviridae*, 12 *Myoviridae*, and 1 *Podoviridae* viruses, while the control-enriched viruses included 16 *Siphoviridae*, 9 *Myoviridae*, 2 *Quimbyviridae*, and 1 *Podoviridae_crAss-like* members. Moreover, we revealed that the CHB-LF-associated gut multi-kingdom signatures were tightly interconnected, suggesting that they may act together on the disease. Finally, we showed that the microbial signatures were effective in discriminating the patients from healthy controls, suggesting the potential of gut microbiota in the prediction of CHB-LF and related diseases.

**Discussion:**

In conclusion, our findings delineated the fecal bacteriome, mycobiome, and virome landscapes of the CHB-LF microbiota and provided biomarkers that will aid in future mechanistic and clinical intervention studies.

## Introduction

Despite the introduction of vaccines that have reduced the risk of hepatitis B virus infection, the global impact of hepatitis B remains profound. In 2019, the number of individuals testing positive for hepatitis B surface antigen (HBsAg) worldwide was approximately 296 million, with a global CHB infection rate of around 3.5% and an estimated 820,000 deaths (Jeng et al., 2023). According to a World Health Organization report, there are about 1.2 million new cases of chronic hepatitis B infection each year, with an estimated 1.1 million deaths in 2022 (WHO, 2024; Seto et al., 2018). Moreover, prolonged HBV infection can lead to various complications, including liver fibrosis, cirrhosis, and hepatocellular carcinoma (HCC) (Nguyen et al., 2020). CHB-related liver fibrosis (CHB-LF) is a common complication for CHB patients as the disease progresses. Liver fibrosis involves an excessive accumulation of extracellular matrix proteins, which is a characteristic of most types of chronic liver diseases (Bataller and Brenner, 2005). Advanced liver fibrosis can result in portal hypertension, cirrhosis, and liver failure, leading to a significant decline in quality of life and potentially even death (Hernandez-Gea and Friedman, 2011; Campana and Iredale, 2017).

Accumulating evidences indicate that the gut microbiota is playing a key role in chronic liver diseases. Studies have shown that the alterations in the gut microbiome were observed in liver cirrhosis (Qin et al., 2014), alcoholic liver disease (Dubinkina et al., 2017), non-alcoholic fatty liver disease (NAFLD) (Shen et al., 2017), autoimmune hepatitis (Wei et al., 2020), early HCC (Ren et al., 2019), and hepatic encephalopathy (Bajaj et al., 2012). In cirrhosis, various microbial metabolites and bacterial endotoxins in the gut can enter the portal circulation through the intestinal wall barrier and reach the liver, activating the liver immune system and inducing inflammation (Usami et al., 2015). Specifically, when the liver is compromised, the lipopolysaccharides (LPS) by gut microbiota can exacerbate the development of hepatitis (Kim et al., 2024). On the other hand, the liver secretes bile acids into the gut via the biliary tract, which affects the composition and abundance of gut microflora (Ridlon et al., 2013; Ridlon et al., 2015). Animal studies have found that a high-fat diet can lead to changes in gut microbiota, which promote the progression of NAFLD through different pathways such as metabolism and immunity (Chiu et al., 2017; Velázquez et al., 2019). For chronic hepatitis B, Wang et al. found that the dysbiosis of gut microbiota was associated with altered hepatic functions and serum metabolites in CHB patients (Wang et al., 2017), and this phenomenon was also found in the gut microbiota of cirrhotic and/or HCC patients with hepatitis B virus infection (Wei et al., 2013; Liu et al., 2019). Moreover, in addition to bacteria, the gut fungal community (or “mycobiome”) and viral community (or “virome”) also play important roles in certain liver diseases including alcoholic hepatitis and NAFLD (Demir et al., 2021; Lang et al., 2020; Hsu et al., 2021). Based on these efforts, a common issue for most microbiome studies is that they are often based on amplicon sequencing (mainly 16S rRNA gene sequencing) of the fecal samples, which limits the systematic investigation of all gut microbial participators such as fungi and viruses.

In this study, we characterized the gut bacteriome, mycobiome, and virome of 25 patients with CHB-LF and 28 healthy controls by deep whole-metagenome shotgun sequencing of their fecal samples. The associations of CHB-LF-associated multi-kingdom signatures were examined, which might help clarify the role of gut microorganisms in the development of CHB-LF and contribute to potential translational opportunities for the prevention and treatment of CHB and related diseases.

## Methods

### Subject recruitment

Twenty-five patients with CHB-related liver fibrosis were recruited from the Shenzhen Hospital of Beijing University of Chinese Medicine. Diagnosis of CHB-LF was based on (1) the viral hepatitis control guild of China; (2) HBeAg and/or HBV-DNA positive; (3) at least two of the four liver fibrosis indicators were abnormal; (4) hepatic imaging data showed uneven echo of liver parenchyma and widened portal vein diameter; and (5) liver elasticity should be greater than 7.4 kPa as confirmed via transient elastography (Chinese Foundation for Hepatitis Prevention and Control, 2019; Salkic et al., 2014). Patients included should comply with the following principles: (1) never taken antiviral therapy before enrollment; (2) no pharmaceutical microflora regulator and dairy products were taken within 2 weeks before the experiment; (3) strong compliance and consent to participate in the study. Patients with non-HBV liver hepatitis or fibrosis, decompensated cirrhosis, liver cancer, severe cardiovascular or kidney diseases; immune system disorders; hematopoietic diseases were excluded. Patients who were allergic to drugs used in this study, in pregnancy or lactation, or mentally ill persons were also excluded. Twenty-eight healthy subjects recruited from the Dalian Medical University Affiliated Xinhua Hospital were introduced from the previous study (Chen et al., 2022). The inclusion criteria of healthy subjects were: (1) without liver diseases, serious cardiovascular diseases, renal failure, or serious autoimmune diseases, and free from other abnormal liver conditions; (2) without antibiotics, antifungals, antivirals, or probiotics treatment within 1 month before sampling. The detailed information of the phenotypic and clinical characteristics of the CHB-LF patients and healthy controls are summarized in Table 1.

**Table 1:** Characteristics of the CHB-LF patients and healthy controls recruited in this study.

	CHB-LF patients (*n* = 25)	Healthy controls (*n* = 28)	*P*-value
Basic characteristics			
Gender (female/male)	4 / 21	10 / 28	0.129
Age (years)	42.2 ± 8.7	56.5 ± 8.7	<0.001
Body mass index (BMI) (kg/m^2^)	22.1 ± 2.7	23.2 ± 2.8	0.678
HBV DNA level (*10^6^ IU/ml)	1.9 ± 5.6	0 ± 0	<0.001
Liver and kidney functions			
Aspartate aminotransferase (AST) (U/L)	75.4 ± 93.4		
Alanine aminotransferase (ALT) (U/L)	79.1 ± 67.6		
γ-glutamyl transpeptidase (γ-GT) (U/L)	66.5 ± 48.4		
Alkaline phosphatase (ALP) (U/L)	102.5 ± 44.3		
Total bile acids (TBA) (μmol/L)	15.4 ± 26.8		
Total protein (TP) (g/L)	74.3 ± 3.3		
Total bilirubin (TBIL) (μmol/L)	20.2 ± 13.8		
Direct bilirubin (DBIL) (μmol/L)	5.4 ± 6.9		
Indirect bilirubin (IBIL) (μmol/L)	14.9 ± 7.7		
Cholinesterase (U/L)	7921.3 ± 3010.8		
Triglycerides (mmol/L)	1.6 ± 1.4		
Total cholesterol (TCHO) (mmol/L)	5.3 ± 2.2		
High density lipoprotein (HDL) (mmol/L)	1.2 ± 0.3		
Low density lipoprotein (LDL) (mmol/L)	3.3 ± 1		
Urea (mmol/L)	4.8 ± 0.9		
Crea (μmol/L)	77.2 ± 12		
Uric acid (UA) (μmol/L)	377.9 ± 69.7		
Liver fibrosis and ultrasonic diagnosis			
Hyaluronidase (HA) (mg/L)	133.3 ± 223.1		
Type III procollagen (PIIINP) (μg/L)	8.1 ± 5.1		
Type IV collagen (IV-C) (μg/L)	65.3 ± 77.1		
Serum laminin (LN) (μg/L)	96.4 ± 64.9		
Oblique diameter of right lobe of liver (mm)	124.2 ± 9.3		
Internal diameter of portal vein (mm)	10.8 ± 1		
Spleen thickness (mm)	38.5 ± 5.7		
Fat attenuation coefficient (dB/m)	230.3 ± 21.7		
Liver hardness (kPa)	13.5 ± 5.7		
Fibrosis stage (F1/F2/F3/F4)	6/9/5/5		

The data for CHB-LF patients and healthy controls were presented as mean ± SD. *p*-values were calculated by Fisher’s exact test for gender and Student’s *t*-test for other basic characteristics.

### Fecal sample collection and whole-metagenome shotgun sequencing

Fecal samples were collected with sterile feces collection containers and stored in a − 80°C freezer until use. Total DNA from fecal samples was extracted using the TianGen Biotech fecal DNA extraction kit, following the provided instructions, as we have previously done in our earlier study (Lv et al., 2024). DNA concentration and purity were determined by NanoDrop2000 and Qubit 4.0. Total microbial DNA was fragmented using Covaris M220 (Gene Company Limited, China). The fresh genomics DNA samples were mechanically fragmented to ~400 bp with Bioruptor Pico (Diagenode, Belgium). A magnetic beads-based method was used for DNA fragments selection following a standard protocol (Agencourt AMPure XP). Libraries were prepared by using the NEBnext® Ultra™ II DNA 540 Library Prep Kit for Illumina^®^ (New England BioLabs). All libraries were barcoded and pooled to perform whole-metagenome shotgun sequencing at the NovaSeq 6,000 platform (Illumina, USA).

Initial base calling of the sequencing dataset was using the default parameters under the sequencing platform. The raw metagenomic reads for each sample were independently subjected to quality control using fastp (Chen et al., 2018). Fastp processed with the raw sequencing reads by trimming the low-quality bases (*Q* < 30) at the end of reads and filtering ‘N’-containing (> 3 ‘N’) reads, reads contaminated with adapters, or reads that were too short (< 90 bp), resulting in high-quality reads. The human reads were dismissed from the high-quality reads based on their Bowtie 2 (Langmead and Salzberg, 2012) alignment to the human reference genome (GRCh38).

### Analyses of the gut bacteriome, mycobiome, and virome

The gut bacteriome and mycobiome compositions of fecal samples were analyzed using the previous methods (Chen et al., 2022). Briefly, the bacteriome was profiled based on the Unified Human Gastrointestinal Genome (UHGG) database (Almeida et al., 2020), which comprised 204,938 non-redundant genomes from 4,644 gut prokaryotes. For each sample, we aligned high-quality sequencing reads to the UHGG database using Bowtie2. The aligned reads were then normalized to generate species-level abundance. And the gut mycobiome was profiled based on the available fungal genomes from the National Center of Biotechnology Information (NCBI) RefSeq database, which comprised 1,503 fungal genomes from 106 human gut-derived fungal species. For each sample, the high-quality nonhuman metagenomic reads were aligned with fungal genome references to create gut fungal profiles. Reads that matched fungal rRNA/tRNA gene sequences were excluded. To prevent contamination from other gut microbes such as bacteria, archaea, and viruses, the reads mapped to fungal genomes were further aligned against (i) all bacterial, archaeal, and viral sequences from the NCBI NT database and (ii) 4,644 prokaryotic genomes from the UHGG database (Almeida et al., 2020). Contaminating reads identified through this process were removed. The relative abundances of 106 fungal species were calculated for each sample, and the relative abundances at the family and genus levels were determined by summing the abundances of species within the same taxa.

Metagenomic reads from fecal samples were assembled *de novo* using MEGAHIT (Li et al., 2015) with a range of k-mer sizes (--k-list 21,41,61,81,101,121,141). For each sample, contigs with a length > 5,000 bp were selected for viral identification and annotation, following the previously established methods (Guo et al., 2022). The viral contigs were then dereplicated at 95% sequence similarity and over 75% coverage to create a non-redundant catalog. The quality and completeness of the viruses were estimated by CheckV (Nayfach et al., 2020). The gut virome of all samples was profiled based on the non-redundant viral catalog.

### Statistical analyses

Statistical analysis was conducted using the R language.^1^ Principal coordinates analysis (PCoA) was carried out using the R *vegan* (Oksanen et al., 2013) package, based on the Bray-Curtis distance of all samples. Permutational multivariate analysis of variance (PERMANOVA) was performed using the *adonis* function of the *vegan* package, and the *adonis p* was calculated via 1,000 permutations. The method of effect size analysis was referred to as the Wang et al. (2020) study. Random forest models were trained using the R *randomForest* (Liaw, 2015) package (with 1,000 trees) to distinguish between CHB-LF patients and healthy individuals by using the abundance profiles of the differential bacteria, fungi, and viruses. The performance of the random forest models was evaluated using receiver operator characteristic (ROC) analysis which was realized with the R *pROC* (Robin et al., 2011) package. The Wilcoxon rank-sum test was used to assess statistical differences between patients and controls. *p* was adjusted for multiple testing to obtain the *q* using the Benjamini–Hochberg procedure. A *p* (for a single test) or *q* (for multiple testing) less than 0.05 was considered statistically significant. Visualization was performed using R *ggplot2* (Wickham et al., 2016) package. The phylogenetic tree visualization was done using iTOL (Letunic and Bork, 2021).

To explore the relationships among bacterial species, fungal species, and viruses, we conducted a correlation analysis using Spearman’s rank correlation coefficient. For each pair of microbes, a correlation coefficient was calculated based on the relative abundances after adjusting for individuals’ gender, age, and body mass index. Only the inter-microbe correlation coefficients >0.6 (positive correlation) or < −0.6 (negative correlation) were regarded as strong correlations and included for analysis. The correlation network was visualized using Cytoscape v3.8.2 (Su et al., 2014).

## Results

### Alterations of the gut bacteriome in CHB-LF patients

To investigate the gut microbial composition of 25 CHB-LF patients and 28 healthy controls, we obtained a total of 349.8 Gbp of high-quality data (on average, 6.6 ± 1.3 Gbp per sample) based on whole-metagenome shotgun sequencing of their fecal samples. We mapped the metagenomic reads into the UHGG database Needless and generated a gut bacteriome profile of 5,125 bacterial and archaeal taxa, including 24 phyla, 39 classes, 77 orders, 146 families, 512 genera, and 4,327 species.

**Figure 1:**
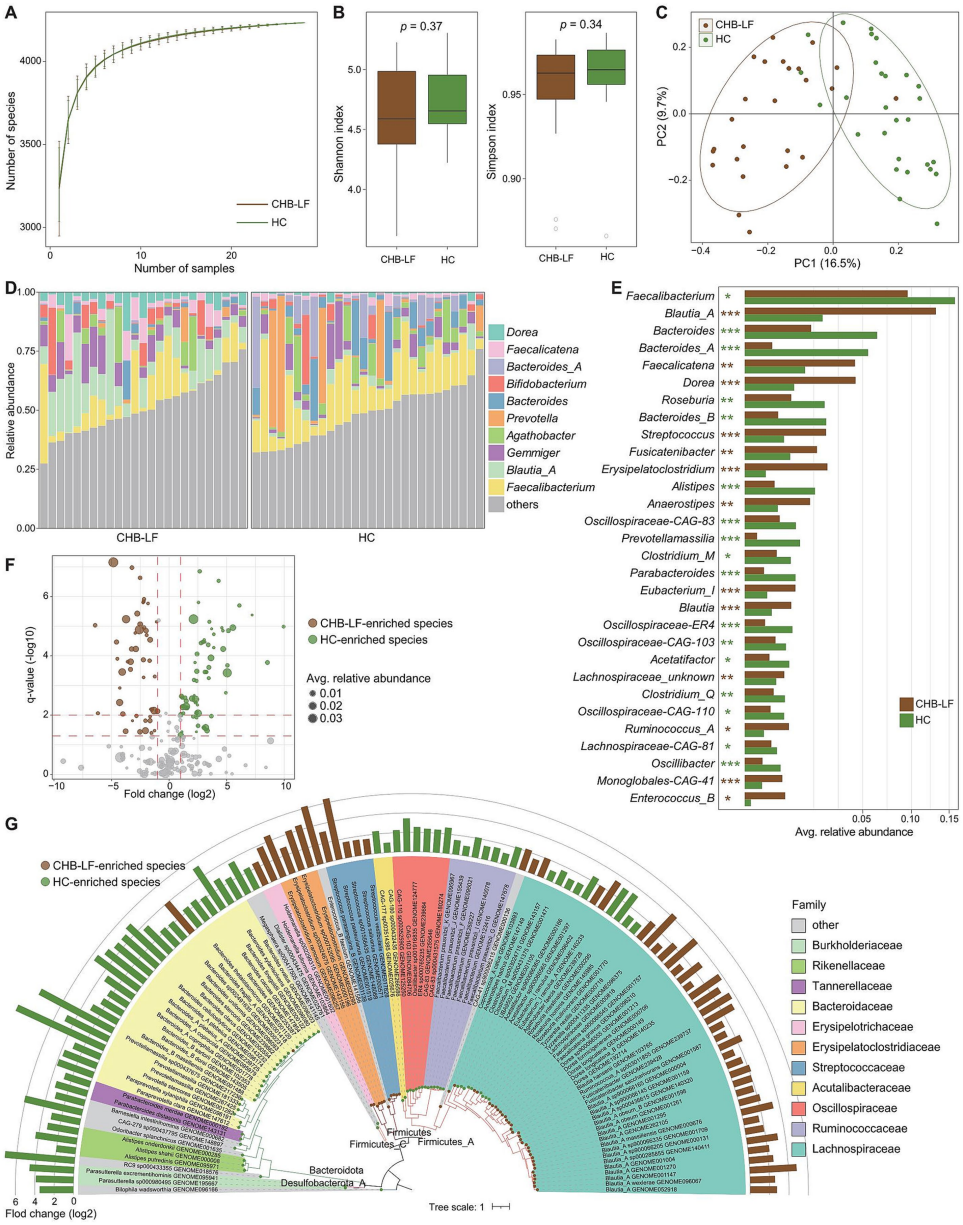
Difference in gut bacteriome between CHB-LF patients and healthy controls. **(A)** Rarefaction analysis of the species richness (estimated by the observed number of species) on each group of samples. The number of species in different groups is calculated based on 30 replacements. **(B)** Boxplot shows the Shannon diversity index (left panel) and the Simpson index (right panel) of gut bacteriome that differ between two groups. **(C)** PCoA analysis of Bray-Curtis distance based on the composition of gut bacteriome, revealing the separations between CHB-LF patients and healthy individuals. **(D)** Composition of gut bacteriome at the genus level. **(E)** Boxplot shows the differential gut bacterial genera when compared between patients and controls. The significance level is calculated based on the Wilcon rank-sum test: *, *q* < 0.05; **, *q* < 0.01; ***, *q* < 0.001. **(F)** Volcano plot shows the fold change vs. *q* for all bacterial species. The X-axis shows the ratio of species abundance in patients compared with that in controls. The Y-axis shows the *q* (−log10 transformed) of the species. **(G)** Detailed information of 110 CHB-LF-associated species. Innermost circle, phylogenetic tree analysis of species based on their genome sequences. The colors in the tree indicate the phylum-level taxonomic assignment of the species. Medium circle: taxonomic assignment of the species at the family level. Outermost circle: barplot shows the fold changes of species abundance in patients compared with that in healthy subjects.

Firstly, we conducted a within-sample diversity analysis of the gut bacteriome at the species level. Rarefaction analysis revealed that the number of observed species was approximately equal between CHB-LF patients and healthy controls when the sample sizes were the same (Figure 1A). Additionally, both the Shannon diversity index and the Simpson index showed no significant differences between the two groups (Wilcoxon rank-sum test *p* > 0.05 for both indexes; Figure 1B). Next, we performed a principal co-ordinates analysis (PCoA) to further investigate the differences in the gut bacteriome between CHB-LF patients and healthy individuals. Clear separations were observed between the two groups (PERMANOVA R^2^ = 11.6%, *p* < 0.001; Figure 1C) indicating a significant distinction in the overall gut bacterial structure between them.

The top 10 most abundant genera in all fecal samples are shown in Figure 1D. Among these genera, *Blautia_A* (average relative abundance 13.1% vs. 2.2% in patients vs. controls, *q* = 3.8×10^−8^), *Faecalicatena* (4.4% vs. 1.3%, *q* = 0.0013), and *Dorea* (4.4% vs. 0.9%, *q* = 2.7e^−6^) were significantly more abundant in the gut bacteriome of CHB-LF patients compared to healthy controls. Conversely, *Faecalibacterium* (9.5% vs. 15.9%, *q* = 0.018), *Bacteroides* (1.6% vs. 6.3%, *q* = 8.2×10^−4^) and *Bacteroides_A* (0.3% vs. 5.7%, *q* = 4.8×10^−5^) were reduce in CHB-LF patients (Figure 1E; Supplementary Table S1). Additionally, 24 low-abundance genera showed significant differences in relative abundance between the two cohorts (Wilcoxon rank-sum test *q* < 0.05). These genera included *Streptococcus*, *Fusicatenibacter*, and *Erysipelatoclostridium* that enriched in CHB-LF patients, as well as *Roseburia* and *Bacteroides_B* that depleted in patients (Supplementary Table S1).

At the species level, 110 species significantly differed between the bacteriome of CHB-LF patients and healthy controls (Wilcoxon rank-sum test *q* < 0.05, |fold change| > 2, and average relative abundance >0.1%). Among these, 48 species were enriched in patients and 62 were enriched in controls (Figure 1F; Supplementary Table S2). The CHB-LF-enriched species were composed of members of *Blautia_A* (*n* = 16 species, including *B. wexlerae*, *B. massiliensis*, and *B. obeum*), *Dorea* (*n* = 5 species, including *D. longicatena* and *D. formicigenerans*), *Streptococcus* (*n* = 4 species, *S. salivarius* and *S. pasteurianus*), *Erysipelatoclostridium* (*n* = 4 species, including *E. ramosum*), and other clades (Figure 1G). Inversely, the control-enriched species were mainly composed of *Bacteroides* (*n* = 11 species, including *B. finegoldii*, *B. ovatus*, *B. stercoris*, *B. thetaiotaomicron*, and *B. uniformis*), *Faecalibacterium* (*n* = 7 species, including several subspecies of *F. prausnitzii*), *Bacteroides_A* (*n* = 4 species, including *B. plebeius_A*, *B. coprocola*, and *B. coprophilus*), and other species.

Finally, we employed HUMAnN3 (Beghini et al., 2021) to profile the microbial functions in all fecal metagenomes, and a total of 406 MetaCyc pathways were comparatively analyzed between HC and CHB-LF subjects. At the pathway level, Shannon index and Simpson were similar between the two cohorts (Supplementary Figures S1A,B). However, the functional composition of the two cohorts was markedly separated on the PCoA plot, consistent with the observations in phylogenetic composition (PERMANOVA R^2^ = 12.1%, *p* = 0.001; Supplementary Figure S1C). Out of 406 pathways, 46 showed significant differences between the two cohorts (with 9 enriched in HC and 37 in CHB-LF subjects, respectively; Supplementary Figures S1D,E; Supplementary Table S3). The pathways enriched in the HC group included L-arginine biosynthesis III (MetaCyc pathway ID: PWY-5154), flavin biosynthesis III (PWY-6168), *β*-(1,4)-mannan degradation (PWY-7456), biotin biosynthesis II (PWY-5005), and L-histidine degradation (HISDEG-PWY and PWY-5030). On the other hand, the pathways associated with CHB-LF included acetylene degradation (P161-PWY), purine nucleobases degradation I (P164-PWY), peptidoglycan biosynthesis (PWY-5265 and PWY-6471), hexitol fermentation (P461-PWY), and formaldehyde assimilation III (P185-PWY).

### Alterations of the gut mycobiome in CHB-LF patients

**Figure 2:**
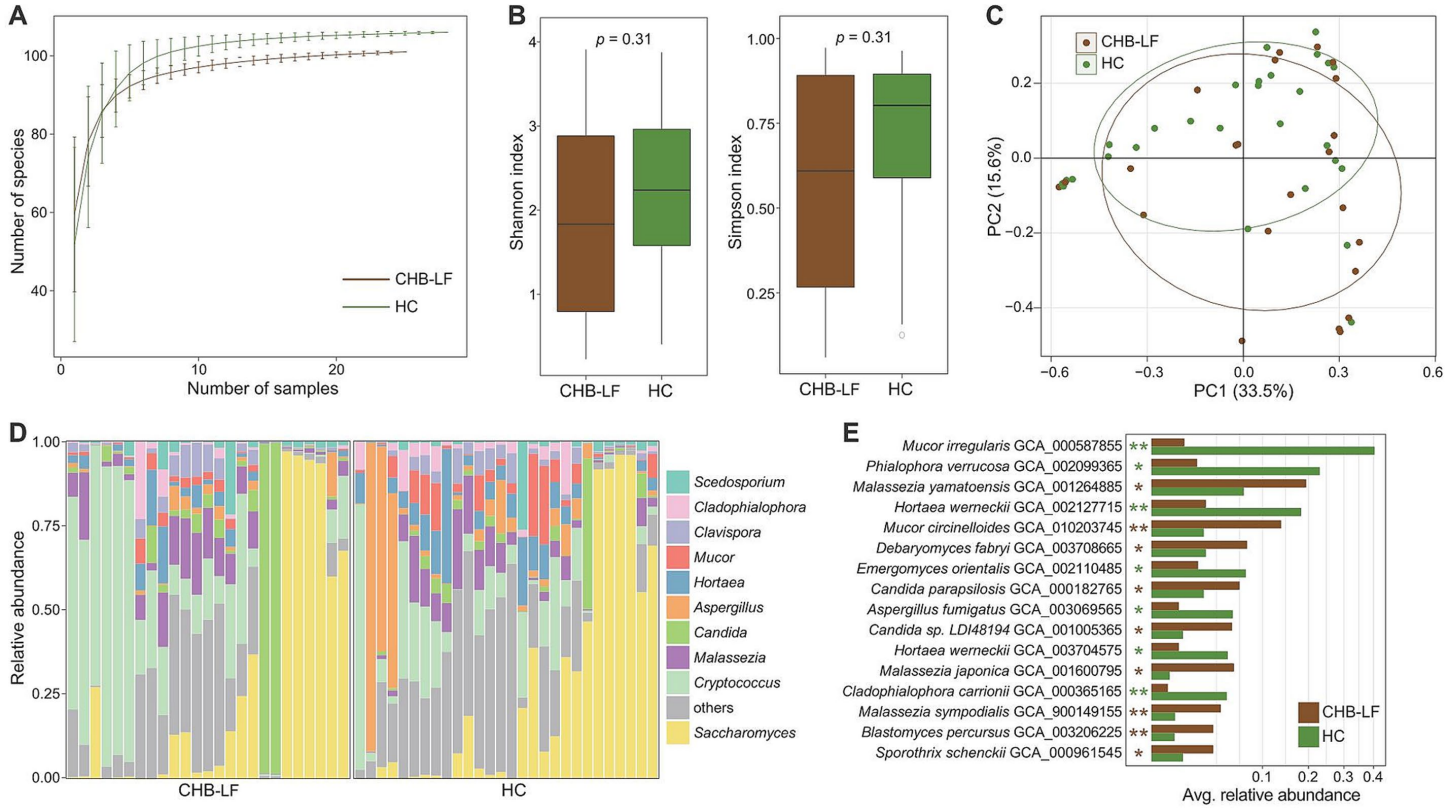
Difference in gut mycobiome between CHB-LF patients and healthy controls. **(A)** Rarefaction analysis of the number of observed species on each group of samples. The number of species in different groups is calculated based on 30 replacements. **(B)** Boxplot shows the Shannon diversity index (left panel) and the Simpson index (right panel) of gut mycobiome that differ between two groups. **(C)** PCoA analysis of Bray-Curtis distance based on the composition of gut mycobiome, revealing the separations between two groups. The location of samples (represented by nodes) in the first two principal coordinates is shown. Lines connect samples in the same group, and circles cover samples near the center of gravity for each group. **(D)** Composition of gut mycobiome at the genus level. **(E)** Boxplot shows the differential gut fungal species when compared between patients and controls. The significance level is calculated based on the Wilcon rank-sum test: *, *q* < 0.05; **, *q* < 0.01.

To explore the variation of gut mycobiome in CHB-LF patients, we profiled the gut fungi of all fecal metagenomes based on the available gut fungal genomes (representing 45 genera and 106 species) and compared their compositions between CHB-LF patients and healthy controls. Rarefaction analysis revealed that healthy controls exhibited a slightly higher level of gut fungal richness compared to CHB-LF patients; however, this difference was not statistically significant (Wilcoxon rank-sum test *p* > 0.05 at the same sample sizes; Figure 2A). Similarly, both the Shannon diversity index and the Simpson index showed no significant difference between the two groups (Figure 2B). Nevertheless, in line with observations in the gut bacteriome, PCoA analysis of the gut mycobiome demonstrated a notable distinction between the CHB-LF and control groups (PERMANOVA R^2^ = 4.7%, *p* = 0.022; Figure 2C).

The top 10 most abundant fungal genera across all fecal samples are illustrated in Figure 2D. None of these genera were significantly enriched in the mycobiome of CHB-LF patients. However, three of them, including *Aspergillus* (average relative abundance 1.7% vs. 10.0% in patients vs. controls, *q* = 0.048), *Hortaea* (3.2% vs. 6.5%, *q* = 0.044), and *Cladophialophora* (1.7% vs. 2.8%, *q* = 0.043), were significantly depleted in patients (Supplementary Table S4). Besides these genera, six low-abundance genera exhibited significant differences in relative abundance between the two cohorts (Wilcoxon rank-sum test *q* < 0.05). Specifically, *Yarrowia*, *Debaryomyces*, *Blastomyces*, and *Stachybotrys* were enriched in CHB-LF patients, while *Phialophora* and *Emergomyces* were enriched in healthy controls (Supplementary Table S4).

At the species level, 16 fungi were identified as differential species between the gut mycobiome of CHB-LF patients and healthy subjects (Wilcoxon rank-sum test *q* < 0.05, |fold change| > 2, and average relative abundance >0.1%; Figure 2E; Supplementary Table S5). Nine species, including several members of *Malassezia* (*n* = 3 species, composing *M. japonica*, *M. sympodialis*, and *M. yamatoensis*), *Candida* (*n* = 2 species, composing *C. parapsilosis* and *Candida* sp. LDI48194), *Mucor circinelloides*, *Debaryomyces fabryi*, *Sporothrix schenckii*, and *Blastomyces percursus* were more abundant in the gut mycobiome of CHB-LF patients. On the other hand, 7 species, including *Mucor irregularis*, *Phialophora verrucosa*, *Hortaea werneckii* (*n* = 2 subspecies), *Emergomyces orientalis*, *Aspergillus fumigatus*, and *Cladophialophora carrionii* were more abundant in healthy subjects.

### Alterations of the gut virome in CHB-LF patients

**Figure 3:**
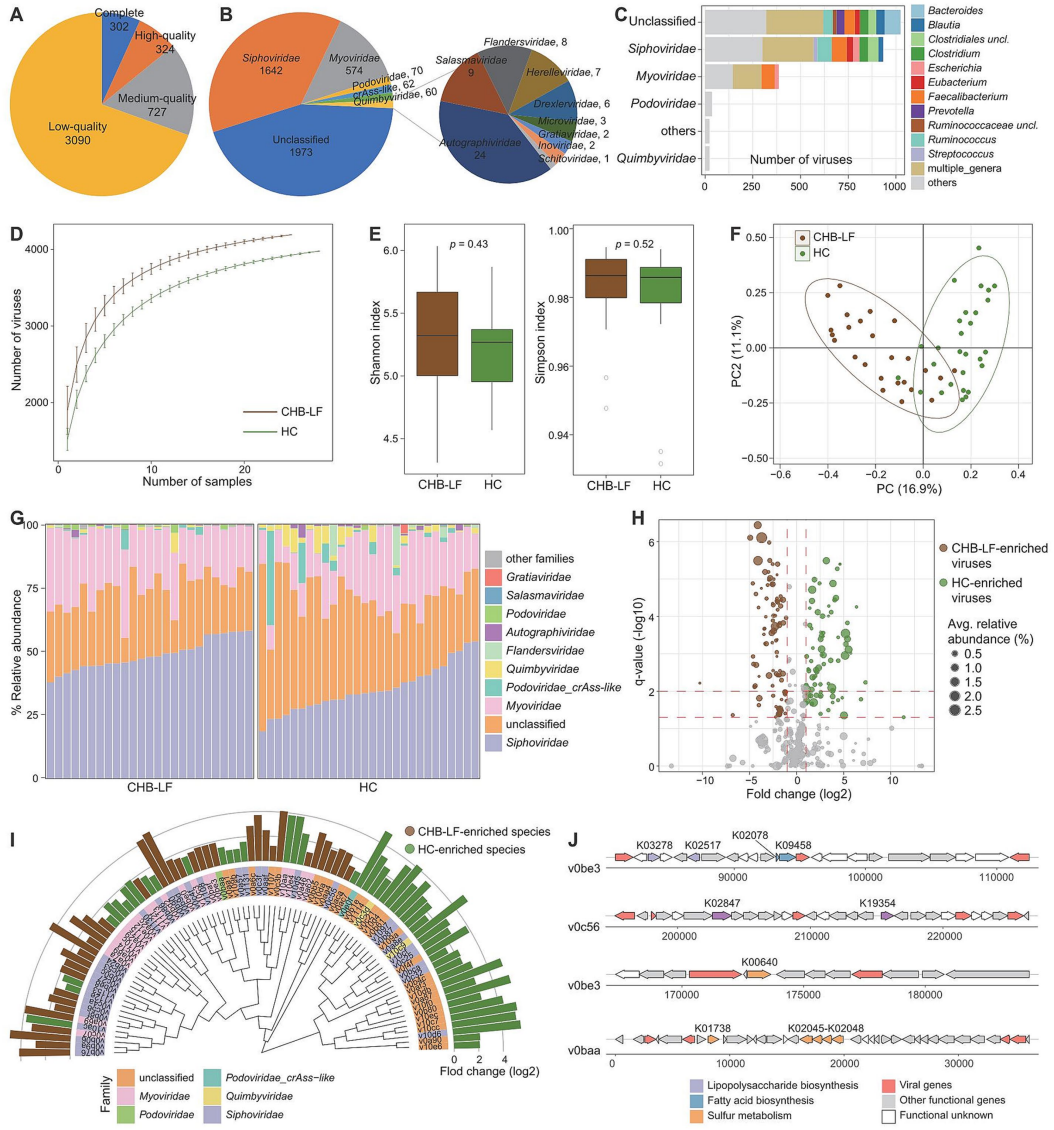
Characteristics of the gut virus catalogue and gut virome. **(A,B)** Pie plot shows the quality **(A)** and family-level taxonomic annotation **(B)** of the non-redundancy virus catalogue. **(C)** The bacterial host assignment of the non-redundancy virus catalogue. **(D)** Rarefaction curve analysis of the number of observed viruses on each group of samples. The number of viruses in different groups is calculated based on 30 replacements. **(E)** Boxplot shows the Shannon diversity index (left panel) and the Simpson index (right panel) of gut virome that differ among two groups. **(F)** PCoA analysis of Bray-Curtis distance based on the composition of gut virome, revealing the separations between two groups. **(G)** Composition of gut mycobiome at the family level. **(H)** Volcano plot shows the fold change vs. *q* for all viruses. The X-axis shows the ratio of viral abundance in patients compared with that in controls. The Y-axis shows the *q* (−log10 transformed) of the viruses. **(I)** Detailed information of 90 CHB-LF-associated viruses. Innermost circle, phylogenetic tree analysis of species based on their genome sequences. Medium circle: taxonomic assignment of the viruses at the family level. Outermost circle: bar plot shows the fold changes of viral abundance in patients compared with that in healthy subjects. **(J)** Genome structure of several viruses contained genes involving lipopolysaccharide biosynthesis and sulfur metabolism.

To explore the gut virome characterization of CHB-LF patients, we identified a total of nonredundant 4,443 viral sequences (minimum length 5,000 bp) from the metagenomically assembled contigs of all 53 fecal metagenomes (see Methods). The length of this gut viral catalogue ranged from 5,000 bp to 538,410 bp, with an average length of 28,241 bp and an N50 length of 65,779 bp. Based on the estimation result by CheckV (Nayfach et al., 2020), 14.1% of these viruses were evaluated as complete or high-quality viral genomes, and 16.4 and 69.5% of them were medium- and low-quality viruses, respectively (Figure 3A) 55.6% of 4,443 viruses could be robustly assigned to a known viral family. Consistent with the observations in the previous gut viral databases (Gregory et al., 2020; Camarillo-Guerrero et al., 2021; Nayfach et al., 2021), *Siphoviridae* (37.0%, *n* = 1,642) and *Myoviridae* (12.9%, *n* = 574) were the most dominate genera the classified viruses, and other representative viruses included members of *Podoviridae* (1.6%, *n* = 70) and *Podoviridae_crAss-like* (1.4%, *n* = 62), and *Quimbyviridae* (1.4%, *n* = 60) (Figure 3B). Additionally, 54.6% (2,425/4,443) of the viruses could be assigned into one or more bacterial hosts based on their homology to genome sequences or CRISPR spacers (see Methods). The most common identifiable bacterial hosts were members of Firmicutes (mainly *Ruminococcus*, *Faecalibacterium*, *Eubacterium*, *Clostridium*, *Blautia*, and *Streptococcus*), Bacteroidota (mainly *Bacteroides* and *Prevotella*) (Figure 3C).

Rarefaction analysis showed that the CHB-LF patients had a significantly higher gut viral richness than the healthy controls at the same sample size (Figure 3D). However, both the Shannon diversity index and Simpson index were not different between the two groups, suggesting that their within-sample viral diversities were similar (Wilcoxon rank-sum test *p* > 0.05, Figure 3E). In addition, PCoA analyses of the gut virome at the species level showed that the overall structure of viromes of CHB-LF patients and healthy controls were significantly separated, with an effect size R^2^ of 11.7% and PERMANOVA *p* < 0.001 (Figure 3F).

The family-level composition of all samples was shown in Figure 3G. Four families had significantly differed in the relative abundances of gut virome between CHB-LF patients and healthy controls, including *Siphoviridae* (average relative abundance 48.7% vs. 35.2% in patients vs. controls, *q* = 4.7×10^−6^) and *Salasmaviridae* (0.14% vs. 0.07%, *q* = 2.6×10^−4^) that enriched in patients and *Quimbyviridae* (0.7% vs. 2.8%, *q* = 2.6×10^−4^) and *Autographiviridae* (0.2% vs. 0.6%, *q* = 0.028) that enriched in controls (Supplementary Table S6). Using Wilcoxon rank-sum test, we identified 90 viruses with significant differences in abundances between two groups (*q* < 0.05, |fold change| > 2, and average relative abundance >0.1%; Figure 3H). 39 of these 90 differential viruses were enriched in CHB-LF patients and 51 of them were depleted. Taxonomically, the CHB-enriched viruses included 18 *Siphoviridae*, 12 *Myoviridae*, and 1 *Podoviridae* members, while the control-enriched viruses included 16 *Siphoviridae*, 9 *Myoviridae*, 2 *Quimbyviridae*, and 1 *Podoviridae_crAss-like* members (Figure 3I; Supplementary Table S7).

We predicted a total of 13,586 protein-coding genes from 90 CHB-LF-associated viruses and annotated the functions of 21.2% of these genes based on the KEGG database. Most of the annotated genes had involved in genetic and environmental information processing and typical viral functions, apart from them, 184 genes were viral auxiliary metabolic genes (AMGs). Exploration of the AMGs generated two interesting findings:

(1) we found that two CHB-LF-enriched viruses (v0be3 and v0c56) encoded the lipopolysaccharide (LPS) synthetases, suggesting that these viruses might involve in the biosynthesis of LPS which leads to disease; (2) two CHB-LF-enriched viruses, v0be3 and v0baa, contained genes that might participate in sulfur metabolism in the human gut (Figure 3J). These findings suggested the potential metabolic capabilities of the CHB-associated viruses in the human gut virome.

### Multi-kingdom gut microbial signatures and their correlations with clinical metrics

**Figure 4:**
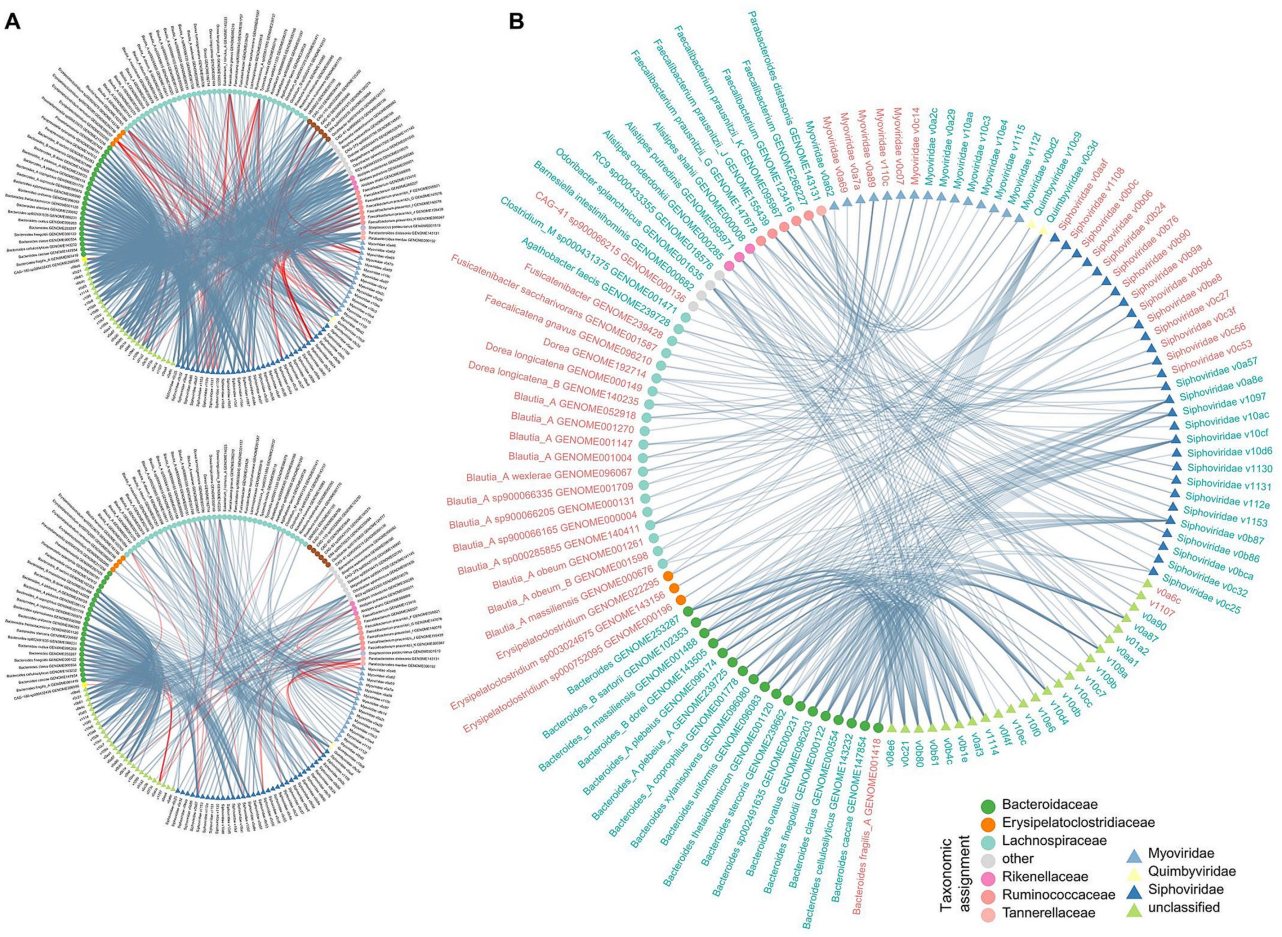
Correlation analysis among multi-kingdom microbial signatures. **(A)** Network showing the multi-kingdom microbial correlations within CHB-LF patients (upper panel) and healthy controls (bottle panel). **(B)** Sharing correlations between CHB-LF and control networks. Gut bacteria and viruses are colored based on their family-level taxonomic assignment. The microbial names are colored by their enrichment in CHB-LF patients (red) and healthy controls (green).

To explore the relationship among CHB-associated gut bacterial, fungal, and viral signatures, we performed a correlation analysis between 110 differential bacterial species, 16 differential fungal species, and 90 differential viruses. Using an absolute Spearman correlation coefficient threshold of 0.6, we identified 1,077 co-abundance relationships between bacterial species and viruses within the CHB-LF patients and 429 relationships within the healthy subjects (Figure 4A), suggesting that the gut bacteriome and virome were tightly connected regardless of disease. Notably, specific correlations were significant, including a strong negative correlation between Alanine aminotransferase (ALT) levels and *Collinsella* GENOME279141 (Spearman’s rho >0.9, *q* < 0.05), as well as between Type IV collagen (IV-C) levels and *RUG420* genome GENOME208505 (Supplementary Figure S2). However, no strong co-abundance correlation was found between gut fungi and bacteria/viruses in both groups. Moreover, 349 bacterium-virus relationships were shared between the two groups, which generated a correlation network spanning 54 bacteria and 70 viruses (Figure 4B). Viruses belonging to Siphoviridae were primarily connected to the bacteria, especially members of Bacteroidaceae (*Bacteroides*, *Bacteroides_A*, and *Bacteroides_B*) and *Blautia_A*, while some *Myoviridae* viruses were frequently connected to the *Faecalibacterium* species; the roles of these viruses and bacteria need further study.

### Prediction of CHB-LF using microbial signatures

**Figure 5:**
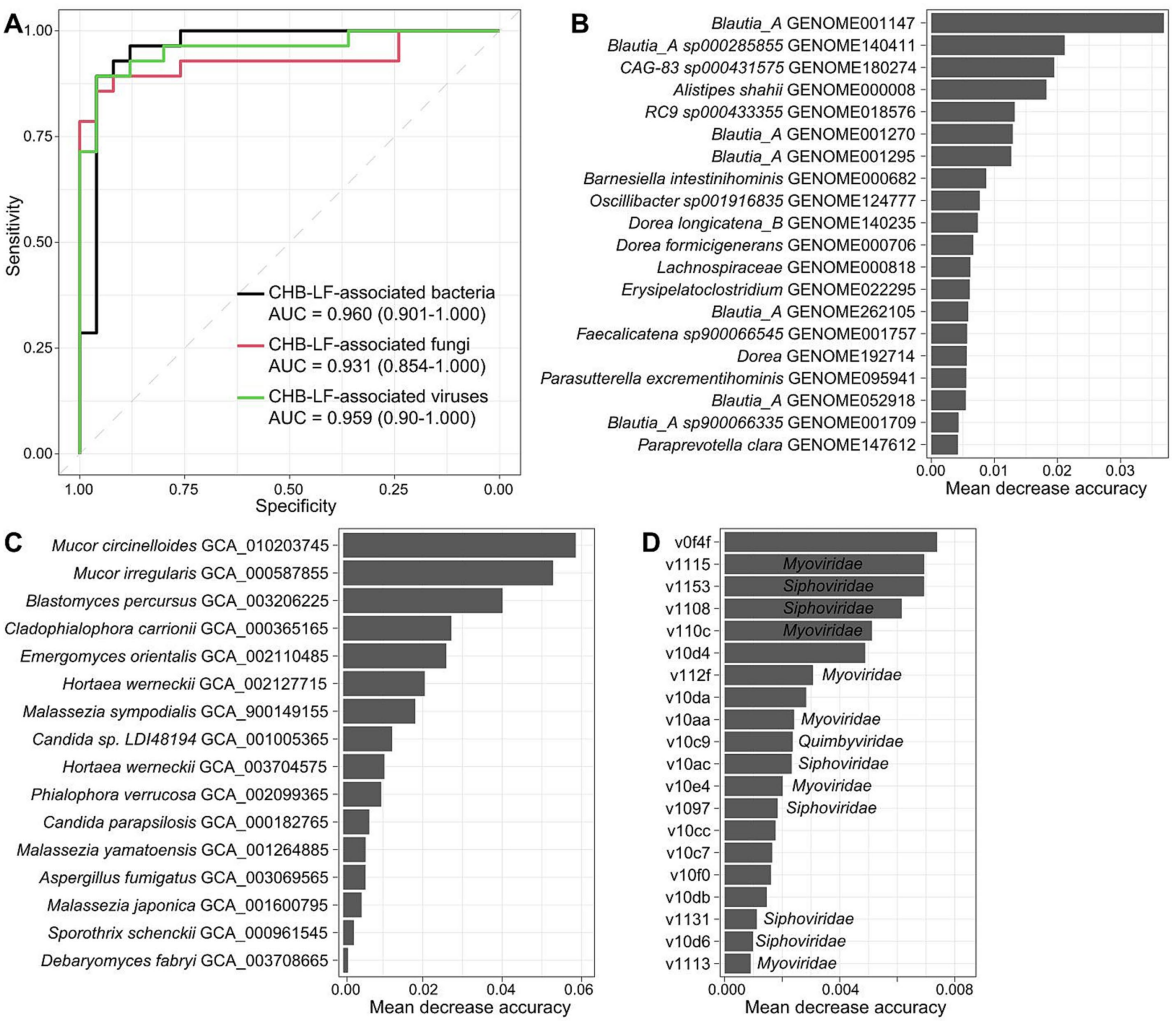
Classification of CHB-LF patients and healthy controls by gut multi-kingdom signatures. **(A)** ROC analysis for classification of disease status using the gut bacterial, fungal, and viral signatures. **(B–D)** The 20 most discriminant bacterial **(B)**, fungal **(C)**, and viral signatures **(D)** in the models for classifying patients and controls. The bar lengths indicate the importance of the microbes.

Finally, we evaluated the performance of multi-kingdom signatures of the gut microbiome to identify CHB-LF status using the random forest model. The models that were trained based on the bacterial, fungal, and viral signatures obtained the discriminatory powers of the area under the receiver operator characteristic curve (AUC) of 0.960, 0.931, and 0.959, respectively (Figure 5A), suggesting high predictability of disease status based on these signatures. Some CHB-LF-associated bacteria, including several members of *Blautia_A*, *Alistipes shahii*, and *Barnesiella intestinihominis* had the highest importance score in the random forest model (Figure 5B). Likewise, the most important CHB-associated fungi were *Mucor circinelloides*, *Mucor irregularis*, *Blastomyces percursus*, and *Cladophialophora carrionii* in the model (Figure 5C), and the most important viruses included the unclassified v0f4f and members of *Myoviridae* (e.g., v1115, v110c) and *Siphoviridae* (e.g., v1153, v1108) (Figure 5D). The application of these signatures in disease diagnosis deserves further exploration.

## Discussion

Though the gut bacterial richness and diversity had no statistical difference between CHB-LF patients and healthy controls, there were significant differences in the structure of the gut bacterial community between them. Specifically, patients were enriched in some species belonging to *Blautia_A*, *Faecalicatena*, *Dorea*, *Streptococcus*, and *Erysipelatoclostridium*. *Blautia* is widely present in the mammalian gut with potential probiotic properties (Liu et al., 2021). As one of the most dominators in the human gut microbiota, the increased abundance of *Blautia* species may positively correlate with some systematic disorders such as bowel symptoms (Brunkwall et al., 2021), irritable bowel syndrome (Biagi et al., 2011), preeclampsia (Lv et al., 2022), and NAFLD (Shen et al., 2017). Inversely, several studies also showed that the composition of gut *Blautia* significantly decreased in patients with chronic liver disease and HCC and was negatively associated with cirrhosis (Chen et al., 2021). The present study demonstrated that several *Blautia_A* species, including *B. wexlerae*, *B. massiliensis*, and *B. obeum*, were highly distributed in CHB-LF patients. Based on this information, however, the potential correlation between *Blautia* and liver diseases is still unclear. Gut levels of *Dorea*, especially the CHB-LF-enriched *D. longicatena* and *D. formicigenerans*, were potential risk factors for obesity and other chronic disorders (Liu et al., 2017; Del Chierico et al., 2017). Also, the *Streptococcus* and *Erysipelatoclostridium* species were potential opportunistic pathogens that positively related to many human disorders including liver cirrhosis (Qin et al., 2014; Stadlbauer et al., 2020). On the other hand, similar to previous research findings (Wang et al., 2017; Wasfy et al., 2024), compared with CHB-LF patients, the gut bacteriome of healthy subjects showed increases in diverse *Bacteroides* (e.g., *B. thetaiotaomicron*, *B. uniformis*), *Bacteroides_A* (e.g., *B. plebeius_A*, *B. coprophilus*), *Faecalibacterium* (mainly *F. prausnitzii*), and *Roseburia* species. Members of *Bacteroides* played essential roles in the human gut with capacities in the decomposition of complex carbohydrates (e.g., animal polysaccharides) and production of bioactive molecules (e.g., B-complex vitamins) (Costea et al., 2018; Engevik et al., 2019), while *Faecalibacterium* and *Roseburia* species were important SCFA producers in the gut ecosystem with anti-inflammatory effects (Machiels et al., 2014). This is consistent with the differential pathways identified in our functional analysis (e.g., PWY-5005: biotin biosynthesis), a finding also observed by Wang et al. (2017). Taken together, these findings suggested considerable gut bacterial dysbiosis in CHB-LF patients and would enhance our understanding and interpretation of the etiology of this disease.

Of the gut mycobiome, a representative finding was the increase of *Malassezia* (i.e., *M. japonica*, *M. sympodialis*, and *M. yamatoensis*) and *Candida* (e.g., *C. parapsilosis*) species in CHB-LF patients compared with the healthy controls. Consistent with the findings of Chen et al. (2011) and Guo et al. (2010) culture study, we observed a higher quantity and diversity of Candida species in the fecal samples of HBV patients compared to healthy individuals. *Malassezia* is a commensal yeast that is usually resident in the human skin, and the overgrowth of some *Malassezia* members in the human gut had been reported to promote gut inflammation and cancers (Limon et al., 2019; Aykut et al., 2019; Das et al., 2021). *Candida* is one of the predominant fungi in the human gut, it contains many opportunistic pathogenic species that can invade the body and cause infections in the mucosa, viscera, and central nervous system (Lockhart, 2014). The enrichment of gut *Candida* was frequently reported in human diseases including IBD (Sokol et al., 2017), autoimmune diseases (Yadav et al., 2022; Chen et al., 2022), and primary sclerosing cholangitis (Rühlemann et al., 2020). Also, agree with our results in the gut mycobiome of CHB-LF patients, a recent study found that the plasma anti-*C. albicans* IgG was increased in patients with NAFLD and advanced fibrosis, suggesting that these patients may have increased systemic immune response to *Candida* species (Demir et al., 2021). On the contrary, several taxa including the *Aspergillus* genus were depleted in the mycobiome of CHB-LF patients. *Aspergillus* can produce a large number of secondary metabolites, and its decrease may indicate that the fungal community has weakened its function on the host (Vesth et al., 2018); further studies are needed to validate this notion.

At the virome scope, we found that the viral richness of CHB-LF patients was significantly increased compared with that of healthy controls, suggesting that these patients may have a higher viral load in their gut. *Siphoviridae* had enriched in the gut virome of CHB-LF patients (Clooney et al., 2019), the same phenomenon was also observed in previous studies of IBD patients, but however, the opposite trend was found in patients with polycystic ovary syndrome or osteoarthritis (Chen et al., 2022; Huang et al., 2022). At the species level, we identified 39 and 51 viruses that enriched in the viromes of CHB-LF patients and healthy controls, respectively. These viruses can serve as CHB-LF-associated viral signatures for future studies. In particular, 70 of the 90 viruses were correlated with the CHB-LF-associated gut bacteria. Many CHB-LF-enriched viruses had been correlated with some *Blautia* species, and similar, many control-enriched viruses were related to the *Bacteroides* species; these findings were consistent with the enrichment of *Blautia* and reduction in Bacteroides in the gut bacteriome of CHB-LF patients. These results suggest that these viruses may depend on the gut bacteria to act in disease. In addition, the other CHB-LF-associated viruses (*n* = 20) may act independently of the gut bacteriome, and their function needs to be further investigated.

Although it is outside the scope of this study to fully investigate viral functions, we specifically noted that 2 CHB-LF-enriched viruses encoded the AMGs that participate in LPS biosynthesis. Virus-encoded enzymes LPS synthetases were also enriched in the gut virome of patients with rheumatoid arthritis (Mangalea et al., 2021), which may be implicated in the modifications of bacterial antigenicity and fitness in the gut community (Rodriguez-Valera et al., 2009). The elevation of LPS also contributes to the progression of hepatitis to a certain extent (Kim et al., 2024). Besides, 2 CHB-LF-enriched viruses encoded several AMGs participated in sulfur metabolism. Virus-associated organosulfur and inorganic sulfur metabolism are prevalently distributed in the human and environmental systems with important implications for human health (Kieft et al., 2021; Kieft et al., 2021). Therefore, the enrichment of these viruses and functions in CHB-LF patients highlighted the importance of broad exploration of the gut viral functions in the liver and related diseases.

This study also has several limitations. First, the sample size is relatively small. Second, the study did not include cases of CHB without liver fibrosis, nor did it collect longitudinal samples. Additionally, due to the difficulty of breaking down fungal cell walls with conventional methods and the limitations of the gut fungal database, we were unable to fully reconstruct the composition of gut fungi. Finally, because we used DNA sequencing, we could only partially reconstruct the composition of DNA viruses and were unable to obtain information on RNA viruses. To address these limitations, we plan to take several key steps in our future research. First, we aim to utilize larger and more comprehensive datasets to enhance the reliability and generalizability of the biological signals we have identified. Following this, we intend to conduct in-depth mechanistic studies by isolating and culturing the bacteria, fungi, or viruses discovered in our research. These studies will be complemented by animal experiments to validate their roles and explore their underlying mechanisms. Besides, including patients with CHB without liver fibrosis would provide a more precise understanding of the relationship between CHB and the gut microbiome, and we plan to consider this population in our future research. Additionally, based on these mechanistic insights, we will select specific microorganisms for clinical intervention studies to assess their effectiveness in practical applications. We believe that these interconnected plans will provide a solid foundation for future mechanistic research and clinical applications.

## Conclusion

In this study, we especially focused on the gut microbial characteristics in patients with CHB-related liver fibrosis, which has not yet been investigated comprehensively by previous studies. Based on deep whole-metagenome shotgun sequencing of fecal samples from 25 CHB-LF patients and 28 controls, we systematically characterized the gut bacteriome, mycobiome, and virome of CHB-LF. We revealed that there were significant differences in the abundance of 110 bacterial species, 16 fungal species, and 90 viruses between the two groups, which may facilitate further mechanistic studies of CHB-LF and related diseases.

## Data Availability

All data generated or analyzed during this study are included in this published article and its Supplementary information files or are available from public repositories. The raw sequencing data for the samples are made available in the European Nucleotide Archive (ENA) under the study identifiers PRJEB79318.

## Footnotes

^1^ https://www.r-project.org/
