# Supplementary figures and images for: Metagenome-based characterization of the gut bacteriome, mycobiome, and virome in patients with chronic hepatitis B-related liver fibrosis
Source: Front Microbiol. 2024 Oct 25;15:1449090. doi: 10.3389/fmicb.2024.1449090 (PMC11543496; doi:10.3389/fmicb.2024.1449090)

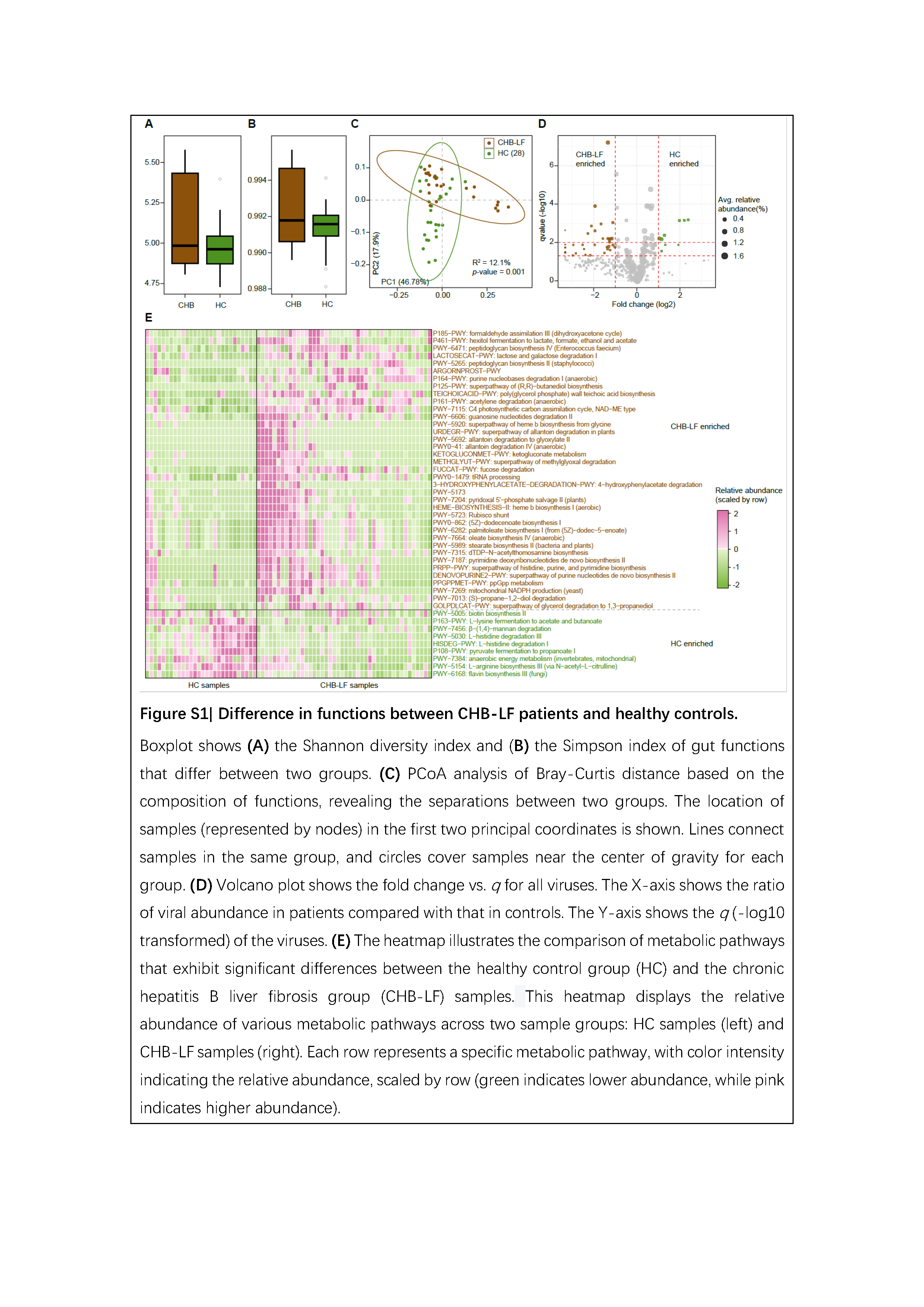

Supplement: Supplementary file 8 [file Image_1.tif]

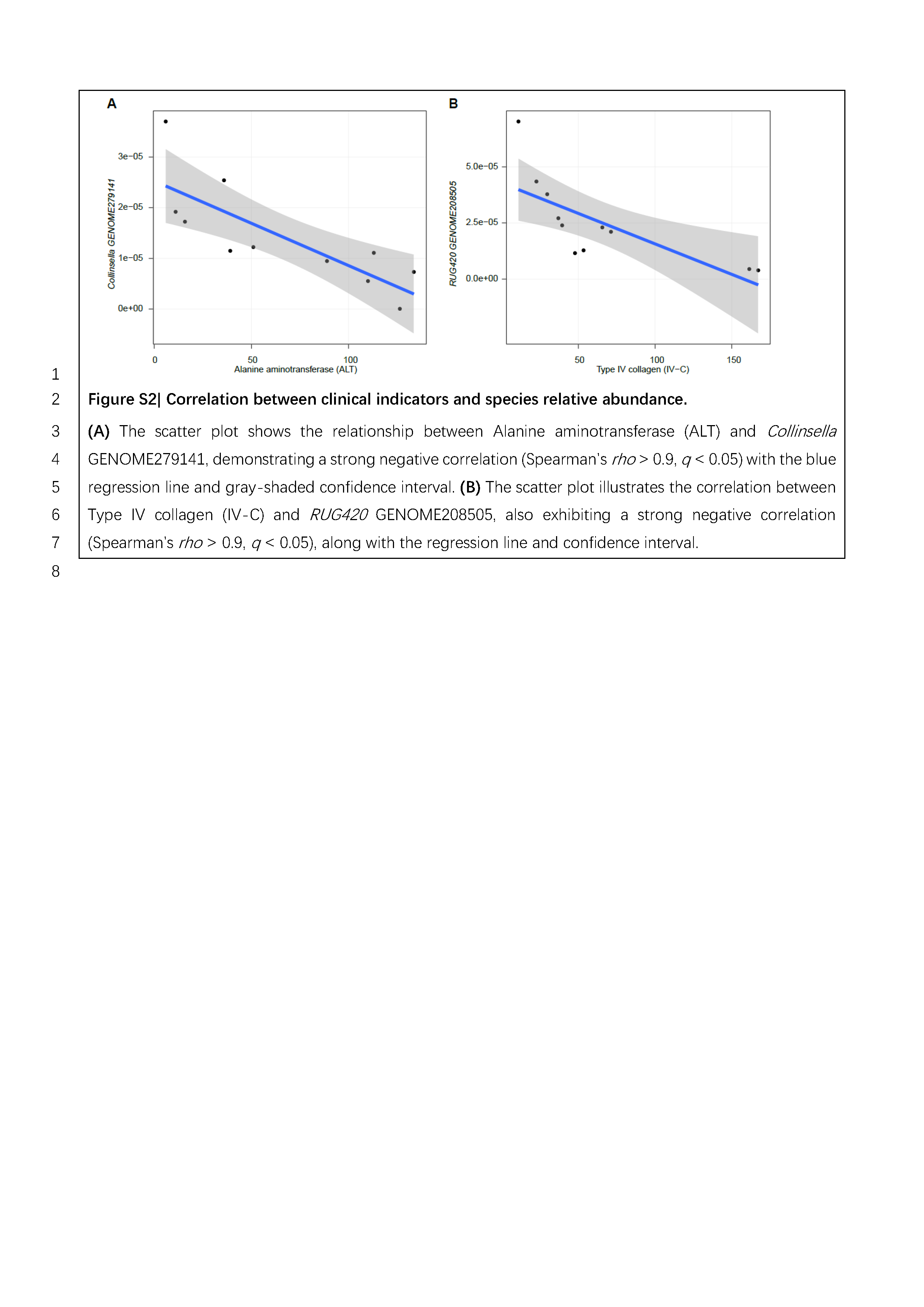

Supplement: Supplementary file 9 [file Image_2.tif]
